# Lifelong leukocyte telomere dynamics and survival in a free‐living mammal

**DOI:** 10.1111/acel.12417

**Published:** 2015-11-02

**Authors:** Jennifer Fairlie, Rebecca Holland, Jill G. Pilkington, Josephine M. Pemberton, Lea Harrington, Daniel H. Nussey

**Affiliations:** ^1^ Institute of Evolutionary BiologyUniversity of EdinburghEdinburghEH9 3FLUK; ^2^ Institute for Research in Immunology & CancerUniversité de MontréalMontrealQCCanadaH3T 1J4

**Keywords:** early‐life environment, longitudinal, mortality, natural selection, Soay sheep, telomere length

## Abstract

Telomeres play a fundamental role in the maintenance of genomic integrity at a cellular level, and average leukocyte telomere length (LTL) has been proposed as a biomarker of organismal aging. However, studies tracking LTL across the entire life course of individuals are lacking. Here, we examined lifelong patterns of variation in LTL among four birth cohorts of female Soay sheep (*Ovis aries*) that were longitudinally monitored and sampled from birth to death. Over the first 4 months of life, there was within‐individual loss of LTL, consistent with findings in the human and primate literature, but there was little evidence of consistent LTL loss associated with age after this point. Overall, we observed only weak evidence of individual consistency in LTL across years and over the entire lifespan: Within‐individual variation was considerable, and birth cohorts differed markedly in their telomere dynamics. Despite the high levels of LTL variation within the lifetimes of individuals, there remained significant associations between LTL and longevity. Detailed analysis of the longitudinal data set showed that this association was driven by improved survival of individuals with longer LTL over the first 2 years of life. There was no evidence that LTL predicted survival in later adulthood. Our data provide the first evidence from a mammal that LTL can predict mortality and lifespan under natural conditions, and also highlight the potentially dynamic nature of LTL within the lifetimes of individuals experiencing a complex and highly variable environment.

## Introduction

Telomeres are DNA–protein complexes at the ends of linear chromosomes that protect against DNA degradation, fusion, and recognition as a DNA discontinuity or break (De Lange, 2004). In mammals, telomeric DNA is comprised of several kilobase pairs of the hexameric repeat 5′‐(TTAGGG)‐3′ followed by a tract of 3′ ssDNA that is folded back into telomeric dsDNA in a ‘t‐loop’ (De Lange, 2004). The immature germline and highly proliferative cell types express an enzyme called the telomerase reverse transcriptase (TERT) that replenishes telomeric tracts through the reverse transcription of its telomerase RNA component (hTR) (Cech, 2004). Later in development, and in many committed cell lineages, TERT is transcriptionally repressed and telomeres erode with each cell division as a consequence of telomere‐trimming and incomplete DNA replication (Cech, 2004). For normal cells grown in culture, this inextricable erosion eventually results in a DNA damage response, and cells enter a nondividing state called senescence (Doksani & de Lange, 2014). In several animal models, telomere erosion in specific tissues has also been documented during organismal aging, which has led to the notion that TL may affect organismal lifespan as well as cellular lifespan (Vera *et al*., 2012; Lopez‐Otin *et al*., 2013). Indeed, in laboratory murine strains, an increase in telomerase expression ameliorates tissue decline with age and results in an increase in average lifespan (de Jesus *et al*., 2011; Vera *et al*., 2013). Whether short telomeres predict subsequent lifespan and healthspan in organisms outside the laboratory, including humans, and whether cellular TL could have any causal effect on organismal function remain questions under intense current investigation (Monaghan & Haussmann, 2006; Aubert & Lansdorp, 2008).

In human populations, average leukocyte telomere length (LTL) is most commonly measured, as blood cells offer a minimally invasive insight into organismal telomere dynamics. LTL exhibits a biphasic age‐related decline, with rapid loss in early development followed by slower loss in adulthood (Aubert & Lansdorp, 2008; Muezzinler *et al*., 2013), and LTL is correlated with TL measured concurrently in other tissue types (Daniali *et al*., 2013). Thus, LTL has been proposed as both a useful surrogate readout of organismal average telomere length and as a biomarker of aging, although evidence that LTL predicts subsequent mortality and morbidity in later adulthood is very mixed (Aviv, 2008; Mather *et al*., 2011). Our current understanding of human LTL dynamics rely largely on cross‐sectional measurements and on longitudinal follow‐up studies that involve two, or rarely three, repeat samples from the same individuals over a 10‐ to 15‐year period (Mather *et al*., 2011; Benetos *et al*., 2013). Although such follow‐up studies cover only a small fraction of a typical human lifespan (< 20%), one recent study, encompassing four different populations, showed remarkably high correlations across measurements on the same individuals (Benetos *et al*., 2013). These findings suggest that individual variation in LTL may be determined genetically or are influenced by early‐life conditions (Benetos *et al*., 2013). However, there is also compelling evidence that environmental conditions throughout life exert a significant impact on LTL, in particular the evidence linking relatively short LTL and experience of conditions that impose physical or psychological stress (Epel *et al*., 2004; Shalev, 2012). As many authors have acknowledged, understanding the factors driving among‐ and within‐individual variation in LTL demands longitudinal collection of samples across the entire lifespan of the organism in question (e.g. Aviv, 2008; Mather *et al*., 2011; Benetos *et al*., 2013). Our own species' long and continually extending life expectancy means such data sets are currently unavailable, and it remains unclear to what degree telomere dynamics measured in short‐lived rodent models, which express telomerase in most tissues and often possess telomeres considerably longer than our own, can offer insights into longitudinal patterns in longer‐lived mammals. Longitudinally monitored populations of mammals with intermediate lifespans and telomere lengths more similar to humans therefore offer our best current prospect for the study of lifelong telomere dynamics (Davis & Kipling, 2005). Here, we present the first study to measure and examine LTL dynamics across the entire lifespan of a mammal outside of the laboratory.

Alongside the rapid growth of studies of LTL within human medicine and epidemiology, there is a burgeoning current interest in telomere dynamics within the field of evolutionary ecology that is motivated by desire to understand the genetic and environmental causes of variation in life histories and aging among species, populations and individuals under natural conditions (Monaghan & Haussmann, 2006). Most of this work has been conducted in birds and reptiles that, unlike mammals, have erythrocytes that remain nucleated, and thus measure erythrocyte TL (ETL) from blood samples rather than LTL. While it should be noted that the relationship between ETL and LTL is not yet known in nonmammalian vertebrates, recent studies have found significant associations between ETL and either survival or reproductive performance in the wild (e.g. Bize *et al*., 2009; Barrett *et al*., 2013; Bauch *et al*., 2013). At the same time, studies of birds kept under laboratory conditions provide evidence for links between stress and ETL shortening (Haussmann *et al*., 2012; Nettle *et al*., 2015) and ETL in early adulthood predicts subsequent lifespan (Heidinger *et al*., 2012). However, studies of LTL dynamics in mammals other than humans and laboratory rodents remain surprisingly rare, with only a handful of examples from wild mammals (Izzo *et al*., 2011; Beirne *et al*., 2014; Lewin *et al*., 2015).

**Figure 1:**
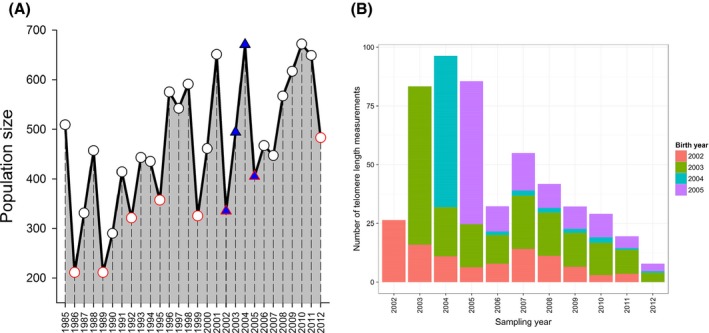
(A) August population size of Soay sheep in Village Bay study area of Hirta, St Kilda. Blue‐filled triangles indicate the four birth cohorts that were sampled in this study; red outlined symbols indicate high mortality ‘crash’ years. (B) Number of relative leukocyte telomere length measures available by capture year, with bar fills differentiating the four different birth cohorts contributing to each total.

The Soay sheep (*Ovis aries*) of St Kilda represent an excellent model system to dissect the factors influencing LTL across the lifespan of a relatively long‐lived mammal. These sheep are isolated, unmanaged and unpredated and thus can be routinely monitored with minimal migration while still under natural selection. Since 1985, those animals resident to the Village Bay area of the island of Hirta in the St Kilda archipelago have been individually marked and monitored longitudinally across their entire natural lives (Clutton‐Brock & Pemberton, 2004). Resident sheep are captured and blood samples are collected at birth in spring (March–April) and each summer (August); thereafter, around 90% of lambs born are caught each year and around 60% of the population are caught in August (Clutton‐Brock & Pemberton, 2004). Importantly, the sheep experience dramatic fluctuations in their environment and population size (Fig. 1A), due to a complex interaction between food limitation, climate and infection (Coulson *et al*., 2001). The vast majority of mortality occurs over‐winter (December–March), and the population dynamic is characterized by periods of low and rising sheep numbers followed by dramatic over‐winter ‘crashes’ in which more than 50% of the population may perish (Fig. 1A; Clutton‐Brock & Pemberton, 2004). The mortality rate of lambs during their first winter can exceed 90% in these ‘crash’ winters and fluctuates widely from year to year outside these crash periods (Fig. S1, Supporting information). Importantly, emigration from the population is rare and searches performed during late winter locate the vast majority of carcasses, so that approximate death dates are known in this population. The Soay sheep are long‐lived (maximum lifespan of 16 and 10 years in females and males, respectively) and females show senescence in survival and fecundity from around 6 years onwards (Colchero & Clark, 2012; Hayward *et al*., 2013).

To evaluate lifelong telomere dynamics in this population, we selected four consecutive birth cohorts of female sheep that experienced different early‐life environmental conditions (2002–2005, Fig. 1A). We chose to focus on females because they have higher survival rates and lower emigration rates than males, and thus offer a broader insight into longitudinal telomere dynamics. We selected these four birth cohorts because, although consecutive, they represent the extremes of environmental conditions experienced by the sheep, encompassing a full shift from crash to recovery to crash (Fig. 1A). We measured LTL in 713 blood samples from 233 different females born between 2002 and 2005, including all available samples from those individuals collected up to and including August 2012 (Fig. 1B). Of the 233 individuals, five or more telomere length measurements were obtained for 54 individuals, and two or more measurements were obtained for 154 individuals. We used a QPCR‐based method to estimate relative leukocyte telomere length (RLTL; see Methods and Data S1) in our samples and set out to test (i) the degree to which age‐related variation in RLTL was driven by within‐individual change, selective mortality of individuals with long or short RLTL, and cohort differences; (ii) the repeatability of RLTL across the lifespan of individuals; and (iii) whether RLTL predicted survival under natural conditions.

## Results

**Figure 2:**
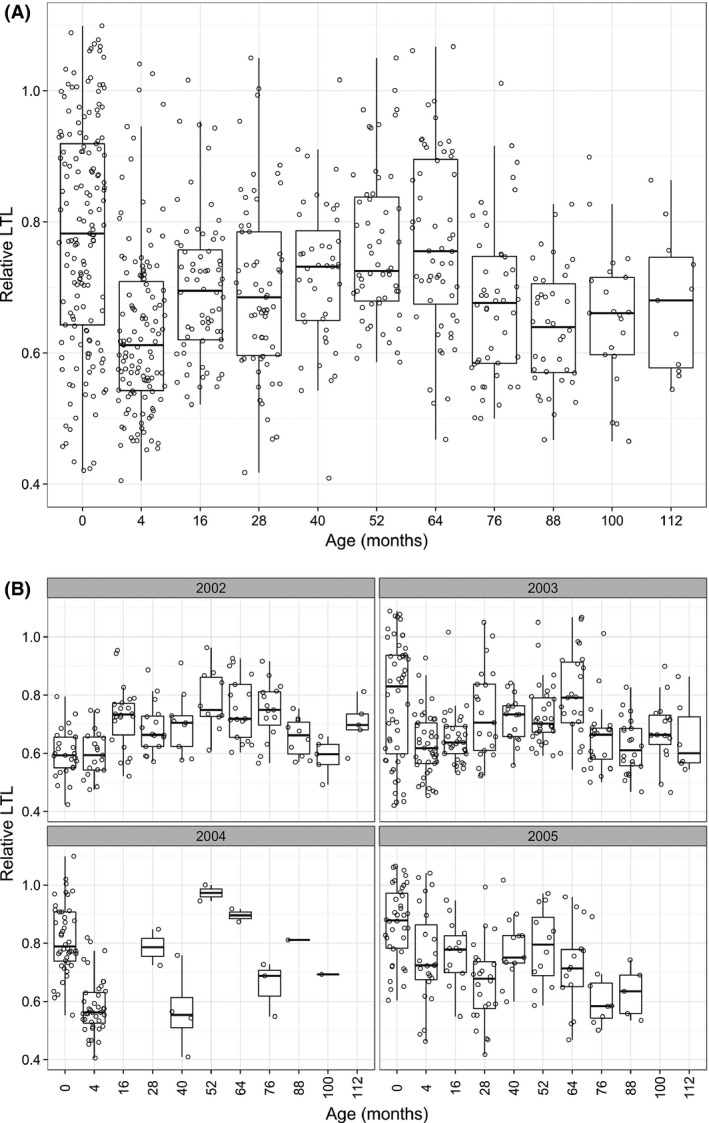
(A) Age‐related variation in relative leukocyte telomere length, illustrated with box and whisker plots for each age group with the raw data points jittered over the top. (B) Age‐related variation in relative leukocyte telomere length (RLTL) varies depending on birth cohort. Box and whisker plots with raw data jittered over top of RLTL by age separately for each birth cohort.

**Figure 3:**
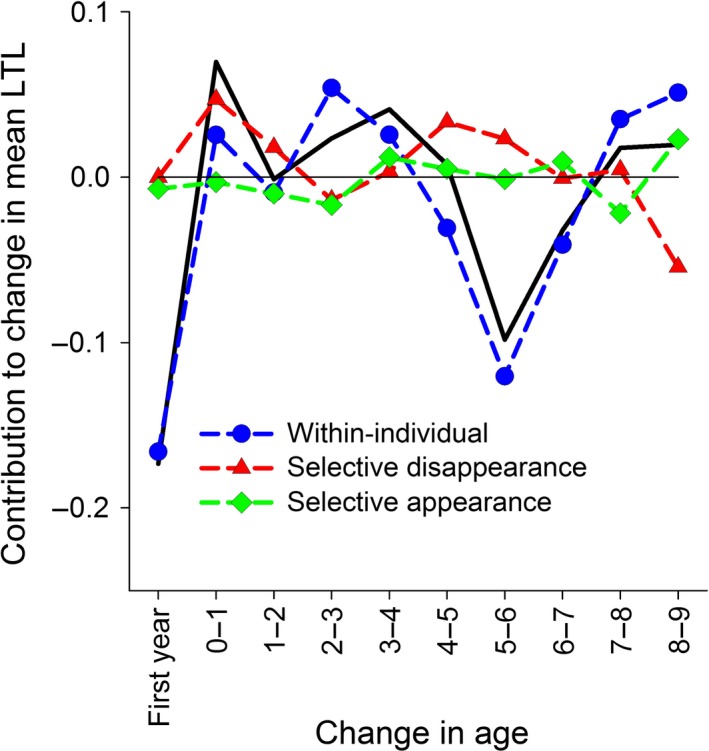
Decomposition of the change in mean relative leukocyte telomere length (RLTL) across age groups into within‐individual and selective components. The black line tracks the absolute difference in mean RLTL across age groups: ‘First year’ denotes change across neonatal and August measures as lamb, ‘0–1’ across August measures as lamb and 1 year old, etc. The other symbols show the contributions of different processes to that difference: Blue circles are within‐individual change, red triangles are selective disappearance effects, green diamonds are selective appearance effects.

We found that the population‐level relationship between mean RLTL and age was complex and driven both by within‐individual changes and selective disappearance effects (Figs 2A and 3). There was a notable decline in RLTL between birth and 4 months of age followed by an increase until 64 months (5 years old), and a secondary decline thereafter (Fig. 2A). There was also considerable overlap in the variance of RLTL across all ages (Fig. 2A). First, we used the approach developed by Rebke *et al*. to decompose changes in mean RLTL across ages into its three constituent components: within‐individual changes in RLTL, differences in the mean RLTL of individuals caught at age *t* but not age *t* + 1 (selective disappearance), and difference in the mean RLTL of individuals caught at age *t* + 1 but not age *t* [selective appearance; see Rebke *et al*. (2010) for full details]. This approach revealed that the decline from birth to 4 months was entirely attributable to within‐individual attrition (Fig. 3). There was also evidence of selective disappearance of individuals with shorter RLTL contributing to increasing means across the first and second year of life (Fig. 3), alongside a suggestion of within‐individual telomere lengthening across the third and fourth year (Fig. 3). The presence of a positive association between telomere length and survival was confirmed by subsequent analyses (see below). The secondary decline in mean RLTL around 6 years of age appeared to be driven by a marked within‐individual decline from 5 to 6 years old (Fig. 3). Despite the wide cross‐sectional RLTL variance across all ages, these analyses reveal strong within‐individual RLTL loss in early life, after which within‐individual change plays a more minor role.

We employed generalized linear mixed‐effects models (GLMMs) of RLTL to address the relationship of RLTL with age and birth cohort and to quantify the degree of among‐individual consistency in RLTL across lifespan after accounting for possible age and cohort effects. GLMMs of RLTL confirmed a very complex relationship between RLTL and age that also exhibited strong birth cohort dependence (Fig. 2B, Table S1, Supporting information). We found that the best‐fitting model for the age dependence of RLTL was to treat each age as a separate factor level: The age‐related variation in RLTL was not as well described by simpler polynomial or threshold age functions of age (Table S1). A model that included age as a factor and its interaction with cohort considerably outperformed models based on age alone (Table S1), demonstrating considerable differences in the pattern of age‐related variation in RLTL among the four birth cohorts (Fig. 2B). Exclusion of twelve females for whom longevity was not known with certainty did not alter these results (Table S2, Supporting information).

**Figure 4:**
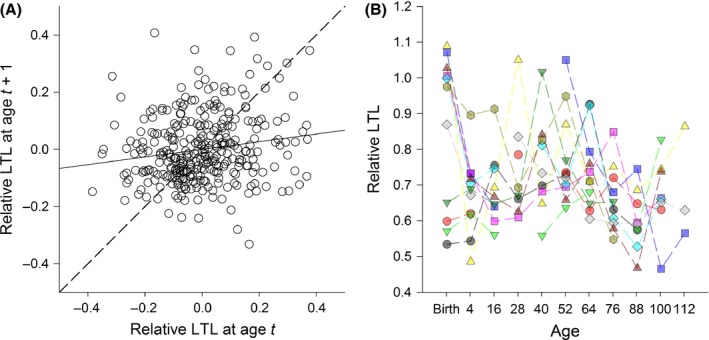
Weak temporal autocorrelation and complex lifelong dynamics in relative leukocyte telomere length (RLTL) in wild sheep. (A) Scatter plot showing of the temporal autocorrelation of RLTL within individual sheep, having corrected for age effects. RLTL values were corrected for age means and then measurements from individuals sampled at consecutive ages were plotted against one another, with the dashed lined indicating a perfect relationship and the unbroken line the actual relationship, which was significant but only weakly positive. (B) Longitudinal telomere dynamics plotted for 11 female sheep that were measured twice as lambs and at least six further times thereafter during their lives. Each color and symbol combination represents a different individual.

We found only weak within‐individual consistency in RLTL across the lifespan, and correlations of measures across subsequent ages were low (Figs 4A and S3, Supporting information). The individual repeatability (see Experimental Procedures) was 0.13, meaning that only 13% of the variance in RLTL can be attributed to consistent among‐individual differences, even after accounting for age, cohort and longevity effects. Furthermore, although there was a significant, positive association between subsequent RLTL measures within individuals, the relationship was weak (Fig. 4A; effect of previous RLTL measurement on current measurement within GLMM including age as fixed factor and capture year as random effect: $\chi_{1}^{2}$ = 9.39, *P* = 0.002, *b* = 0.14 ± 0.05 SE). To further illustrate this lack of among‐individual consistency and dramatic within‐individual variability of RLTL, we have selected 11 females in our sample that were measured in their first year and at least six further times thereafter, and have plotted their RLTL dynamics (Fig. 4B).

Despite the considerable within‐individual variation in RLTL observed in our study population, we found that an individual's mean RLTL across their lifetime was positively associated with their longevity. The addition of a fixed effect for longevity to the best‐fitting GLMM of RLTL including age and cohort (see Table S1) significantly improved model explanatory power (ΔAIC = 6.88 comparing models with and without longevity; effect size of longevity: 0.0064 ± 0.0021 SE). We did not find evidence to suggest that this positive association in longevity differed among cohorts (ΔAIC = −5.29 comparing models with and without interaction between longevity and cohort). Thus, mean RLTL was positively associated with longevity in female Soay sheep, independent of observed age‐ and cohort‐specific variation.

**Figure 5:**
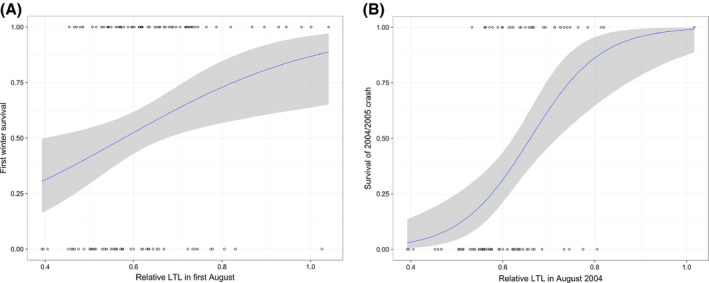
August relative leukocyte telomere length (RLTL) predicts over‐winter survival in young sheep. (A) RLTL in first August plotted against survival of first winter (0 = died, 1 = survived) with logistic regression function plotted as a line and associated confidence intervals as shaded area. (B) RLTL in August 2004 plotted against survival of the population crash over the subsequent winter [details same as (A)].

This positive association between RLTL and longevity detected in our GLMMs was not due to associations between RLTL and survival in later adulthood, but was instead underpinned by survival benefits of long telomeres in young individuals (0–2 years old). In lambs, we found no association between neonatal RLTL and first winter survival ($\chi_{1}^{2}$ = 0.00, *P* = 0.92), but first August RLTL was positively associated with first winter survival ($\chi_{1}^{2}$ = 4.30, *P* = 0.04, *b* = 5.22 ± 2.72 SE, Fig. 5A), independent of cohort. Despite the fact that RLTL declines rapidly between birth and 4 months, we found no association between lamb survival and the change in RLTL between birth and August (neonatal RLTL × August RLTL interaction: $\chi_{1}^{2}$ = 0.10, *P* = 0.78). We also found that RLTL measured in August 2004 predicted survival in the subsequent high mortality winter of 2004/2005, independent of age ($\chi_{1}^{2}$ = 9.8, *P* = 0.002, *b* = 15.41 ± 5.83 SE, Fig. 5B). This association was primarily driven by individuals aged 1 or 2 years (i.e. 2002 and 2003 birth cohorts), as almost all the lambs born in spring 2004 died over their first winter (Fig. S4B). The exclusion of lambs born in 2004 did not alter the effect of RLTL in the model ($\chi_{1}^{2}$ = 5.16, *P* = 0.02, *b* = 12.12 ± 6.17 SE). There was no additional association between 2004/2005 crash survival and RLTL in August 2003 ($\chi_{1}^{2}$ = 2.03, *P* = 0.15). Furthermore, RLTL was not significantly associated with adult survival in models including those aged 3 years or older ($\chi_{1}^{2}$ = 0.10, *P* = 0.71) or those aged 7 or older ($\chi_{1}^{2}$ = 2.60, *P* = 0.10). Finally, using GLMMs of RLTL that considered survival only of animals aged 3 years or older, there was no significant association with longevity ($\chi_{1}^{2}$ = 0.10, *P* = 0.75, *b* = 0.0020 ± 0.0063 SE). Taken together, these data suggest the longevity association is predominantly driven by survival benefits of long RLTL in early life.

## Discussion

To our knowledge, this is the first study to examine lifelong leukocyte telomere dynamics outside of the laboratory and to provide evidence that longer LTL are associated with reduced mortality in a wild mammal. The pattern of age‐related variation we observed at the population level (Fig. 2A) was complex, and our longitudinal data allowed us to demonstrate that this complexity was underpinned by a combination of within‐individual changes and selective mortality. We observed a within‐individual decline in RLTL from birth to 4 months of age consistent with patterns reported in the literature for TL dynamics in the immune cell populations of young humans and primates (Baerlocher *et al*., 2003; Aubert & Lansdorp, 2008). However, more surprising was the observed increase in RLTL between 1 and 5 years of age that appeared to be driven by a combination of selective mortality of individuals with short RLTL and within‐individual increases in RLTL (Figs 3 and 4). Although by far the strongest signal of within‐individual change in RLTL was observed over the first 4 months of life, there also appeared to be an average within‐individual increase in RLTL in early to mid‐adulthood. Although further analysis will be required to rule out statistical and technical artefacts, mean LTL lengthening remains possible. As leukocytes represent a diverse pool of cell types with different telomere lengths, an increase in average RLTL within an individual's lifetime could reflect changes in the proportions of different immune cell types within the lymphocyte pool and could also be influenced by telomerase activity in proliferating lymphocytes (Aubert & Lansdorp, 2008; Weng, 2012). Although there was some evidence for within‐individual declines in RLTL in later life in our study population, there was little evidence of progressive declines in RLTL over subsequent age groups, instead declines were associated with particular years (Figs 3 and S3). This suggests the later‐life declines were more consistent with a response to changes in environmental conditions across years rather than being associated with aging *per se*. Again, further studies will be required to assess to what extent the results are affected by measurement error, compositional changes in the leukocyte populations, and telomere attrition across the leukocyte cell pool on average.

Overall, we observed only weak evidence of individual consistency in RLTL across years and over the entire lifespan: Within‐individual variation was considerable and birth cohorts differed markedly in their telomere dynamics. Previous longitudinal studies of humans, laboratory rodents and birds have typically not reported readily comparable indices of among‐individual consistency for longitudinal telomere length measurements. Here, we used ‘repeatability’, a metric from the animal breeding literature requiring the use of mixed‐effects models, and an estimate of within‐individual autoregression to estimate this effect (see Experimental Procedures). Both methods represent indices that could be readily standardized and compared across studies, and they suggest statistically significant but rather weak individual consistency in RLTL across the lifespan in wild Soay sheep. Comparison of our repeatability and autoregression coefficients to analyses of longitudinal human data is challenging, mainly due to the emphasis in that literature upon correlations between baseline LTL and changes in LTL at follow‐up that are, to some degree at least, an inevitable result of regression to the mean (Verhulst *et al*., 2013). However, a recent study that simply correlated baseline and follow‐up measures and thus avoided issues with regression to the mean found very strong autocorrelation of LTL measures across a 10‐ to 15‐year follow‐up period in four human populations (*r* > 0.90, Benetos *et al*., 2013). The authors argued that their data implied individual LTL rank within a population was largely fixed across the adult life course (Benetos *et al*., 2013). However, other human studies which have directly reported baseline and follow‐up correlations have observed much lower correlations and suggested that LTL may be highly dynamic within‐individuals over much shorter time frames (e.g. Martin‐Ruiz *et al*., 2005; Svensson *et al*., 2011). The reasons for these differences among studies remain to be determined. Previous studies on wild mammals have demonstrated age‐related declines in LTL using cross‐sectional data from Australian sea lions (*Neophoca cinerea;* Izzo *et al*., 2011) and longitudinal data from European badgers *(Meles meles;* Beirne *et al*., 2014), while a cross‐sectional study of spotted hyena (*Crocuta crocuta*) found no association with age but positive associations between LTL and social dominance (Lewin *et al*., 2015). However, ours is the first to estimate individual consistency of LTL across lifespan in a wild mammal, and it is clear that in our Soay sheep population, there is little maintenance of rank differences in LTL among individuals. Importantly, our findings imply that genetic and early‐life environment effects on mean LTL during development will carry across to shape LTL during adulthood only weakly, if at all. However, if environmental and physiological stress influence telomere dynamics as suggested in humans and birds (Shalev, 2012; Monaghan, 2014), perhaps this heightened within‐individual LTL variability is unsurprising. Compared to the benign and largely controlled environments most human or laboratory study populations experience, the Soay sheep live in an immensely variable natural environment and face regular over‐winter food shortages, thermoregulatory challenges, and persistent exposure to a wide array of infectious agents (Clutton‐Brock & Pemberton, 2004).

The pronounced differences in the age‐dependent telomere dynamics of the four different birth cohorts in our study suggest that early environmental conditions can influence lifelong telomere trajectories in natural populations. There is mounting evidence that past psychosocial stress is associated with shorter TL in human children and adults (Shalev, 2012). Similarly, in birds, experimentally or naturally induced stress appears to predict shorter ETL at juvenile or adult life stages (Monaghan, 2014). Differences in exposure to physiological stressors, in particular food availability and exposure to parasites, during early life could explain the differences in telomere dynamics among Soay sheep birth cohorts, as the cohorts do differ dramatically in the environmental conditions they experience. Considering the four cohorts in the present study, we can compare population size (Fig. 1A) and lamb survival rates (Fig. S1) to crudely illustrate the variation in conditions. The year 2003 was characterized by growing but moderate sheep densities with high winter survival rates for lambs born that year (> 85%), suggestive of relatively favorable conditions. Animals born in 2004 experienced relatively high sheep density and a population ‘crash’ over the following winter in which only 6% of lambs survived. Females sampled in Augusts of 2002 and 2005 had experienced ‘crash’ winter conditions *in utero* and, as a result, experienced relatively low sheep densities as neonates and juveniles; however, lamb winter survival rates differed markedly among these two cohorts (96% in 2002 but 53% in 2005). The observed cohort differences in age‐dependent patterns of LTL variation (Fig. 1B) are not readily explained by differences in annual population size or lamb survival rates; we clearly need data from considerably more cohorts and more complete data on pre‐ and postpartum environmental conditions and stress to understand among‐cohort variation in this system. However, the data suggest we should not rely solely on chronological age to explain variation in LTL under natural conditions. Also, as our data included only females, we cannot yet address the important question of whether sex differences affect telomere dynamics, which have been widely reported in the literature (Barrett & Richardson, 2011). The challenge is to understand how sex, early‐life environment, adult environment, and the aging process interact to generate observed telomere dynamics.

The evidence that LTL in humans predicts subsequent mortality remains mixed and has mostly focused on older adults (Mather *et al*., 2011). Similarly, studies in wild birds have identified links between ETL and subsequent survival (Bize *et al*., 2009; Barrett *et al*., 2013). Our study finds support for a positive association between mean RLTL and lifespan in a wild mammal and further demonstrates that this association is mainly driven by RLTL–survival relationships in the first 2 years of life rather than in later adulthood. In the only other lifelong study of comparable detail, ETL in early adulthood of captive zebra finches predicted lifespan more strongly than ETL in later life (Heidinger *et al*., 2012). In female Soay sheep, longer LTL among young animals in the summer preceding a high mortality winter was most predictive of over‐winter survival (Fig. 5B). Importantly, it was summer LTL and not the change in LTL across the previous years that was predictive of survival. LTL measured in August among young sheep may reflect some aspects of an individual's health or immune status that also predicts their ability to withstand the nutritional, thermoregulatory, and infectious challenges over the following autumn and winter. The lack of association between LTL and survival in later adulthood requires further investigation: Female Soays have high survival rates until 6 years of age, limiting our ability to test for such associations to a relatively small number of geriatric sheep aged seven or more (Fig. S1). A much larger sample size is required to definitively establish that LTL does not predict survival or other indices of health and fitness in elderly members of our study population.

In summary, our findings provide the first evidence for positive selection on LTL in a wild mammal. Our data also extend our understanding of the complex associations between blood cell TL, survival, and reproductive fitness across a range of vertebrate taxa. Further longitudinal studies from the wild will help us elucidate how natural selection drives the genetic diversity that underpins variation in TL across the life course under highly variable, challenging environmental conditions.

## Experimental Procedures

### Fieldwork and blood collection

Each spring in the Village Bay study area on Hirta, sheep are caught within a few days of birth, weighed, and marked with unique ear tags, and blood samples are taken. Each August, as many sheep from the study population as possible are rounded up in a series of temporary traps, caught, and processed over a 2‐ to 3‐week period. At capture, 9 mL whole blood is collected into heparin tubes and stored at 4 °C with buffy coat fractions prepared within 24 h of sampling. Blood samples were collected under UK Home Office License number 60/3547. Buffy coat fractions are prepared as follows: Whole blood is spun at approximately 1008g for 10 min and the plasma layer is then drawn off and replaced by the same quantity of 0.9% w/v NaCl solution and spun again at 3000 rpm for 10 min. The intermediate buffy coat layer, comprising mainly white blood cells, is then drawn off into a 1.5‐mL Eppendorf tube and stored at −20 °C until further use.

### DNA extraction

Genomic DNA was extracted from white blood cells using Qiagen Gentra Puregene kit (Catalogue number 158445). DNA concentration and purity was quantified using a Nanodrop 8000 spectrophotometer (Thermo Scientific, Wilmington DE, USA), with acceptable ranges deemed 1.7–2.0 for 260/280 ratios and 2.0–2.2 for 260:230 ratios. If a sample was found to be outside these ranges, it was discarded and re‐extracted, or rejected if further extractions also failed to meet these criteria. DNA integrity was assessed periodically by running a random selection of DNA extracts on agarose gels and checking that clean ‘crowns’ were evident [as recommended by Kimura *et al*. (2010)]. Genomic DNA samples were standardized to a concentration of 10 ng/μL and stored in Tris‐EDTA at −20 °C until further use.

### Telomere length measurement by QPCR

We measured relative leukocyte telomere length (RLTL) using the real‐time quantitative PCR method [following Cawthon (2002)]. This method estimates of the total amount of telomeric sequence present in a sample relative to the amount of a constant copy number control gene. For our telomere reactions, we used primers tel1b (5′‐CGG TTT GTT TGG GTT TGG GTT TGG GTT TGG GTT TGG GTT‐3′) and tel 2b [5′‐GGC TTG CCT TAC CCT TAC CCT TAC CCT TAC CCT TAC CCT‐3′; from Epel *et al*. (2004)].

We used a GeNorm kit (Primer Design, Southampton, UK) to select the best reference gene from a panel of 12 candidate reference genes designed for our study species. Analysis of amplification profiles from this set of genes from several Soay sheep leukocyte DNA extracts was performed using qBase^plus^ (Biogazelle, Gent, Belgium) to rank the candidate reference genes in order of stability of amplification profile, with a highly stable profile deemed to indicate nonvariable copy number of the gene in question. Beta‐2 microglobulin (B2M) was found to be the most stable and was selected here using primers supplied by GeNorm (Catalogue number: HK‐Sy‐Sh‐900).

Reactions were carried out in 384‐well plates, allowing us to include telomeric and B2M reactions on the same plate. An automated liquid handling robot was employed to load the plates (Freedom Evo‐2 150; Tecan, Mannedorf, Switzerland). A master mix was prepared for each primer set containing 5 μL SYBR Green Master Mix (Roche; Catalogue Number: 04913914001). We used the telomere primers at a concentration of 900 nm and the B2M primers at 300 nm in a 10 μL reaction. Each sample of DNA was diluted to 0.1 ng/μL with double‐distilled H_2_O just prior to running the reactions, and 1 μL of this sample is used in each 10 μL reaction. The QPCR assay was performed using a Roche LC480 instrument (10 min at 95 °C, then 50 cycles of 95 °C for 15 s and 60 °C for 60 s). Each sample was conducted in triplicate for each set of primers. In addition to our Soay sheep samples, we included a no template control (water) and a ‘calibrator’ sample in triplicate on each plate. The calibrator is DNA extracted from a large quantity of blood obtained from a single domestic sheep, diluted to the same concentration as our Soay sheep samples. The disassociation curves of telomere and B2M amplification showed a single peak. Furthermore, our no template control samples did not amplify at all for the B2M reactions and had very high Cq values for the telomere reactions (mean Cq across plates: 38.77 ± 1.07 SD) which was far greater than the highest Cq for our samples (26.26), strongly indicating that any amplification was due to primer dimer formation. On each plate, we also included a calibrator sample dilution curve (10× from 10 ng/μL to 0.0001 ng/μL) and checked the Cq values produced declined in a log‐linear fashion (*r* ^2^ > 0.98) before proceeding to analyze a plate.

We used the software LinRegPCR (Ruijter *et al*., 2009) to correct for baseline fluorescence, set a window of linearity for each amplicon group (i.e. separate windows for B2M and telomere reactions), and then to calculate efficiencies and Cq values for each well. Efficiencies for the two reactions were 1.897 (± 0.007 SD) for B2M and 1.644 (± 0.018 SD) for telomeres (see Data S1 for further details).

We calculated relative telomere length (RTL) following Pfaffl (2001), as:

$$\begin{array}{cc} & \text{RLTL}=\left(E_{\mathrm{TEL}}{\;}^{\wedge }\left({\text{Cq}}_{\mathrm{TEL}[\mathrm{Calibrator}]}-{\text{Cq}}_{\mathrm{TEL}[\mathrm{Sample}]}\right)\right)/(E_{B2M}{\;}^{\wedge }\;({\text{Cq}}_{B2M[\mathrm{Calibrator}]}\\ & \;\;-{\text{Cq}}_{B2M[\mathrm{Sample}]})) \end{array}$$

where *E*_TEL_ and *E*_B2M_ are the mean well efficiencies for each amplicon calculated by LinRegPCR, respectively, and Cq_TEL[Calibrator]_ and Cq_TEL[Sample]_ are the mean Cq for telomere calibrator and sample, respectively. Similarly, Cq_B2M[Calibrator]_ and Cq_B2M[Sample]_ are the mean Cq for B2M calibrator and sample, respectively (see Data S1 for further details).

### Statistical methods

We initially examined the distribution of our RLTL data, finding it to be heavily right‐skewed by a very small number of samples with values > 1.2 (eight samples, all above 99th percentile). To normalize our RLTL data set (Fig. S2), and to ensure that results of our statistical analyses were not biased by a small handful of data points at an extreme of the distribution, we elected to remove these eight samples from further analyses. We went on to visualize the population‐level variation in RLTL with age and used two complementary approaches to test the degree to which within‐individual change and selective process shape the population‐level pattern. First, we used the approach developed by Rebke *et al*. (2010) to decompose changes in mean RLTL across ages into its three constituent components: within‐individual changes in RLTL, differences in the mean RLTL of individuals caught at age *t* but not age *t* + 1 (selective disappearance), and difference in the mean RLTL of individuals caught at age *t* + 1 but not age *t* [selective appearance; see Rebke *et al*. (2010) for full details]. Note that as recapture rates of individuals in our study population were < 100% (we typically capture 60–70% of the resident population each August), the contributions from selective disappearance will incorporate both effects of mortality and of animals that simply were not recaptured the following year. We directly tested associations between RLTL and survival and longevity through incorporation of a longevity effect in our models of RLTL and models of survival probability, which we describe below.

Our second approach involved using GLMMs of RLTL to address the form of its relationship with age and whether these trajectories differed among birth cohorts. This approach also allowed us to test the degree of among‐individual consistency in RLTL across the lifespan and any association between an individual's average RLTL and lifespan once age and cohort effects had been accounted for. We began by comparing the fit of a variety of functions describing the relationship between RLTL and age in Gaussian GLMMs including capture year and individual identity as random effects, and birth year (cohort) as a fixed four‐level factor. We considered a null model (no age term fitted), polynomial functions (linear, quadratic, and cubic), a fully factorial age function (each age fitted as separate factor level), as well as a variety of threshold functions. Visual inspection of age‐related variation in RLTL suggested possible inflection points in the relationship in the first few years of life, and then again at around 5 years of age. To test this, we explored models including age functions with both single and double thresholds from 4 to 28 months of age and 40 to 68 months of age. We compared models including these age functions alone and with their interaction with cohort using AIC values. The model with the lowest AIC has the best fit to the data, with a difference in AIC of 2 units being approximately equivalent to a statistically significant (i.e. *P* < 0.05) difference in model fit based on a log‐likelihood test. Having established the best‐fitting age function and whether or not it differed between cohorts, we next tested for independent associations between longevity and RLTL by assessing whether the addition of longevity (as a fixed covariate) or its interaction with cohort improved model fit. Sixteen of the 232 ewes with RLTL measurements could not be assigned a longevity with confidence, and so model comparison of the age function was rerun using this slightly smaller data set (664 observations from 216 ewes) to ensure this did not alter the final model, before the longevity effects were tested.

To determine the relative consistency of RLTL among individuals over the entire lifespan, we calculated ‘repeatability’ from our GLMMs following standard approaches within quantitative genetics (Falconer & Mackay, 1996). The repeatability of RLTL is the ratio of the individual random variance component to the total random variance estimated in our final GLMM, either with or without fixed effects in the model. To estimate the degree to which RLTL at one time point was predicted RLTL at the previous sampling point (i.e. birth for measurements of lambs in August and previous August for all other measurements), we fitted a GLMM of RLTL which included age as a fixed factor, capture year as a random factor, and the individual's previous RLTL measurement as a fixed covariate. This slope of the latter term represents the temporal autoregressive function for RLTL, having corrected for variation associated with age and year of measurement.

To examine the association between RLTL and longevity in more detail, we broke major mortality events across the lifespan of our study animals into three components: (i) lamb first winter mortality, which varies dramatically among years in our study population (Fig. S1B); (ii) survival of the 2004/2005 winter crash, over which around 50% of the study population perished; (iii) adult mortality, which is generally low in females from 3 to 6 years of age but increases with age from 7 years onwards (Colchero & Clark, 2012). For lambs, we ran generalized linear models (GLMs) of whether or not an individual survived (0 = died, 1 = survived) with a binomial error distribution, including cohort as a factor and testing effects of neonatal and first August RLTL and their interaction. For the 2004/2005 crash, we fitted similar model, this time considering RLTL in Augusts 2003 and 2004 (the two measures prior to the crash). For adults, we fitted separate models of survival of the winter following RLTL measurement for individuals aged 3 years or more and 7 years or more, including age (linear covariate) and birth year (factor) in all models. We reran our final GLMM of RLTL using only data from females measured aged 3 years or more, retesting for effects of longevity. All models were run within the r statistical package version 3.2.0 (R Development Core Team, 2008), and GLMMs were run using the ‘lmer’ package.

## Author contributions

This study was conceived and planned by LH, DHN, JF, and RH. Telomere assays were conducted by JF and RH and statistical analyses by DHN. Samples and field data were collected by JGP and JMP. All authors contributed to the writing of the manuscript.

## Funding info

This work was funded by a BBSRC fellowship (BB/H021868/1) and BBSRC responsive mode grant (BB/L020769/1) to DHN, and a Wellcome Trust grant to LH. The long‐term study on St Kilda was supported by NERC responsive mode grants to JMP and others.

## Conflict of interest

None declared.

## Supporting information


**Fig. S1** Mortality dynamics in the study area. (A) Death per year/age class; (B) Proportion of lambs dying in their first winter.
**Fig. S2** Distribution of relative leukocyte telomere length data with and without > 1.2 values.
**Fig. S3** Decomposition of changes in relative leukocyte telomere length by year.
**Fig. S4** Histograms of samples sizes by cohort in survival analyses for (A) lambs in first year of life and (B) samples from August 2004.
**Fig. S5** Correlation between telomere length measurements by TRF and QPCR.
**Table S1** Model comparison of GLMMs including all relative leukocyte telomere length data.
**Table S2** Model comparison of GLMMs including relative leukocyte telomere length data from females with reliable longevity records.
**Data S1** Supplementary methods.
