# Supplementary material for: Lifelong leukocyte telomere dynamics and survival in a free‐living mammal
Source: Aging Cell. 2015 Nov 2;15(1):140–8. doi: 10.1111/acel.12417 (PMC4717268; doi:10.1111/acel.12417)
Supplement: Supplementary file 1 — Fig. S1 Mortality dynamics in the study area. (A) Death per year/age class; (B) Proportion of lambs dying in their first winter. Fig. S2 Distribution of relative leukocyte telomere length data with and without > 1.2 values. Fig. S3 Decomposition of changes in relative leukocyte telomere length by year. Fig. S4 Histograms of samples sizes by cohort in survival analyses for (A) lambs in first year of life and (B) samples from August 2004. Fig. S5 Correlation between telomere length measurements by TRF and QPCR. Table S1 Model comparison of GLMMs including all relative leukocyte telomere length data. Table S2 Model comparison of GLMMs including relative leukocyte telomere length data from females with reliable longevity records. Data S1 Supplementary methods. [file ACEL-15-140-s001.docx]

**Supplementary on-line material for:**

Life-long leukocyte telomere dynamics and survival in a free-living mammal

by Fairlie *et al*.

**Supplementary Methods.**

*Intra-plate quality control and calculation of relative telomere length*

We used the software LinRegPCR (Ruijter *et al.* 2009) to correct for baseline fluorescence, set a window of linearity for each amplicon group (i.e. separate windows for B2M and telomere reactions), and then to calculate efficiencies and Cq values for each well (see Supplementary methods for further information). Across plates, we set constant fluorescence (Nq) thresholds that were within the window of linearity for our amplification curves: 2.594 for B2M and 2.636 for telomeres. We used the mean efficiency across wells within a plate for each amplicon group, excluding outliers (outside the 5^th^ and 95^th^ percentiles), as our estimates of reaction efficiency (as recommended by [Ruijter *et al.* 2009](#_ENREF_23)). We excluded a sample from further analysis if the standard deviation across its triplicate Cq values for either amplicon group was greater than 5% of the mean Cq for that sample. We also excluded any sample if the standard deviation across the triplicate well-specific efficiencies for either amplicon was greater than 5% of the overall mean efficiency for that amplicon group.

The reaction efficiencies differed between our B2M and telomere reactions (mean efficiencies across all the plates eventually run: B2M = 1.897 +/- 0.007 SD; Telomere: 1.644 +/- 0.018 SD). Assuming constant efficiencies across amplicons (as in the so-called “delta-delta” method originally used by Cawthon 2002) when they differ can bias qPCR results, so we calculated relative telomere length (RTL) using a method that did not assume consistent efficiencies (Pfaffl 2001), as follows:

RLTL = (E_TEL_ ^ (Cq_TEL[Calibrator]_ – Cq_TEL[Sample]_)) / (E_B2M_ ^ (Cq_B2M[Calibrator]_ – Cq_B2M[Sample]_))

Where E_TEL_ and E_B2M_ are the mean well efficiencies for each amplicon calculated by LinRegPCR, respectively, and Cq_TEL[Calibrator]_ and Cq_TEL[Sample]_ are the mean Cq for telomere calibrator and sample, respectively. Similarly, Cq_B2M[Calibrator]_ and Cq_B2M[Sample]_ are the mean Cq for B2M calibrator and sample, respectively.

We ran all available DNA extractions from female Soay sheep born in our study area between 2002 and 2005. Only 10 samples out of 713 failed our intra-plate quality controls (see above; <2% of samples) and since this number was so low we simply excluded the samples from our analyses and did not re-run them, leaving 703 RLTL measurements from 232 different females available for analysis.

*Inter-plate variation with QPCR and validation of QPCR versus Southern blot methods*

We assessed inter-plate variation of our QPCR method by selecting DNA extracts from 48 Soay sheep leukocyte samples (all females born 2002-2005) from across a range of ages and running them as described in the Experimental Procedures section on three replicate plates over two days. Intra-plate sample quality control as described above: triplicate Cqs had to fall within 5% of mean and each well’s efficiency had to fall within 5% of plate mean efficiency for that amplicon group, or the sample was rejected. Five sample triplicates failed these criteria across the three plates from five different samples, and these samples were removed from further analyses. We calculated mean Cqs and RTL (as described above) for each of the remaining 43 samples on each plate. We then calculated the mean and variance for each sample across the three plates. From the sample variances we derived a pooled variance for each method, as follows:

$$s_{p}^{2}= \frac{\sum_{i=1}^{k} \left( n_{i}-1 \right)s_{i}^{2}}{\sum_{i=1}^{k} \left( n_{i}-1 \right)}$$

where *i* is the sample (of k = 43 samples), n_i_ is the number of plates for each sample (i.e. 3) and s^2^_i_ is the variance within those samples. We calculated a pooled coefficient of variance as the square root of the pooled variance divided by the mean across the groups. This gave the following values: mean Cq B2M = 28.34, pooled CV = 0.69%; mean Cq telomere = 21.20, pooled CV = 1.22%; mean RTL = 1.22, pooled CV = 10.01%.

We also tested the correlation between RLTL measures obtained using our QPCR methodology and measures obtained using Southern blots of terminal restriction fragments (TRFs) which are regarded by many as the gold standard approach to telomere length measurement. For TRF, 4μg of genomic DNA from each sample selected was digested it at 37^o^C overnight with the restriction enzymes *RsaI* and *HinfI* in a total reaction volume of 50ul. The following day digested samples were loaded into a 0.4% agarose gel (25cm x 15cm) flanked by lambda DNA ladder markers and digested calibrator sample. The gel was run over 2 nights at 45V. The next morning the gel was placed firstly in depyrination buffer (0.25M HCl) for 30 mins, then denatured with 0.5 M NaOH/1.5 M NaCl for 30 mins, and lastly placed in neutralization solution (1.5 NaCl, 0.5 M Tris pH7.5) for 2x 20 mins, with gentle shaking after each solution step. The DNA samples from the gel were then transferred onto a nylon membrane overnight using typical southern transfer in 20x SCC (saline-sodium citrate). The following day the membrane was UV-crosslinked and prehybridized in church buffer for 30 mins. A telomeric oligonucleotide ([CC TAA]^3^) and small amount of lambda ladder were labelled separately with 32P yATP and 32P α-dATP respectively and added to the hybridizing church buffer containing the membrane. The membrane was left to probe at 37°C for 5 hours to overnight. After being washed in 1xSCC, 0.1%SDS and exposed to a phosphorimager screen for at least 3 hours, the membrane was then captured on a phosphorimager. We used TeloTool (Gohring et al 2013) software to quantify the resulting telomeric smears. In total we obtained good quality TRF smears for 26 Soay sheep samples from animals captured in August 2010 for which we also had QPCR-based RLTL measurements. Mean LTL varied between 21.7 and 31.2kb in these samples. The correlation coefficient among QPCR and TRF LTL measurements was 0.58, and is illustrated in Figure S5.

**References:**

Gohring J, Fulcher N, Jacak J , Riha K (2013). TeloTool: a new tool for telomere length measurement from terminal restriction fragment analysis with improved probe intensity correction. *Nucleic Acids Res*. **42**, e21.

Pfaffl MW (2001). A new mathematical model for relative quantification in real-time RT-PCR. *Nucl. Acids Res.* **29**.

Ruijter JM, Ramakers C, Hoogaars WMH, Karlen Y, Bakker O, van den Hoff MJB , Moorman AFM (2009). Amplification efficiency: linking baseline and bias in the analysis of quantitative PCR data. *Nucl. Acids Res.* **37**.

Figure S1. (A): Number of female deaths per year in Village Bay study area on St Kilda categorised by age class, with deaths per year in four years from which study cohorts were drawn highlighted inset, (B) Proportion of lambs surviving their first winter per year with crash years highlighted in red and study cohorts in blue triangles.


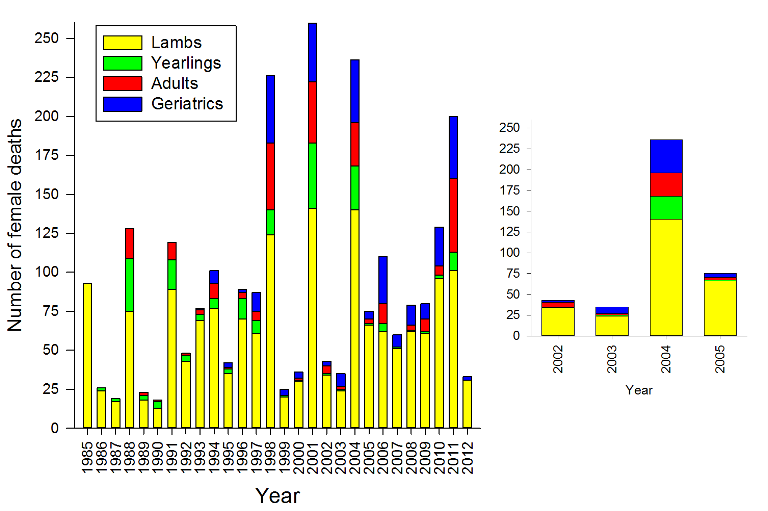

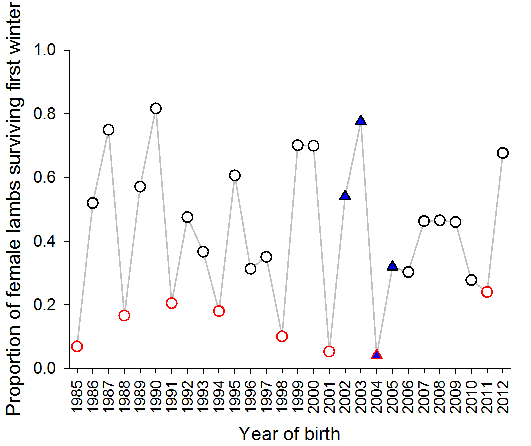


(B)

(A)

Figure S2. Frequency distributions of RLTL data from Soay sheep females on St Kilda with (left panel) and without (right panel) values greater than 1.2.


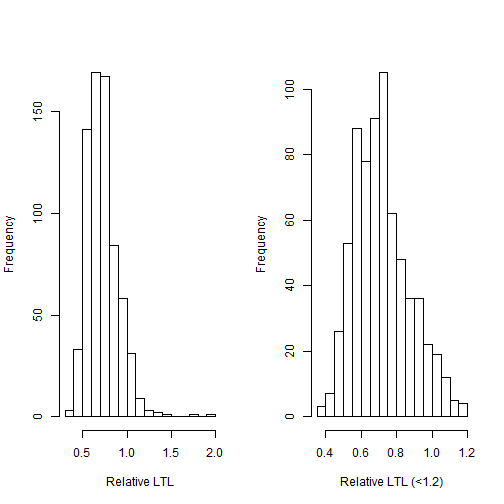


Figure S3. Decomposition of the change in mean RLTL across years into within-individual and selective components, excluding new born lambs from calculations. The black line tracks the absolute difference in mean RLTL across consecutive years (i.e. “02-03” denotes change in mean LTL from August 2002 to August 2003). The other symbols show the contributions of different processes to that difference: blue circles are within-individual change, red triangles are selective disappearance effects, green diamonds are selective appearance effects.


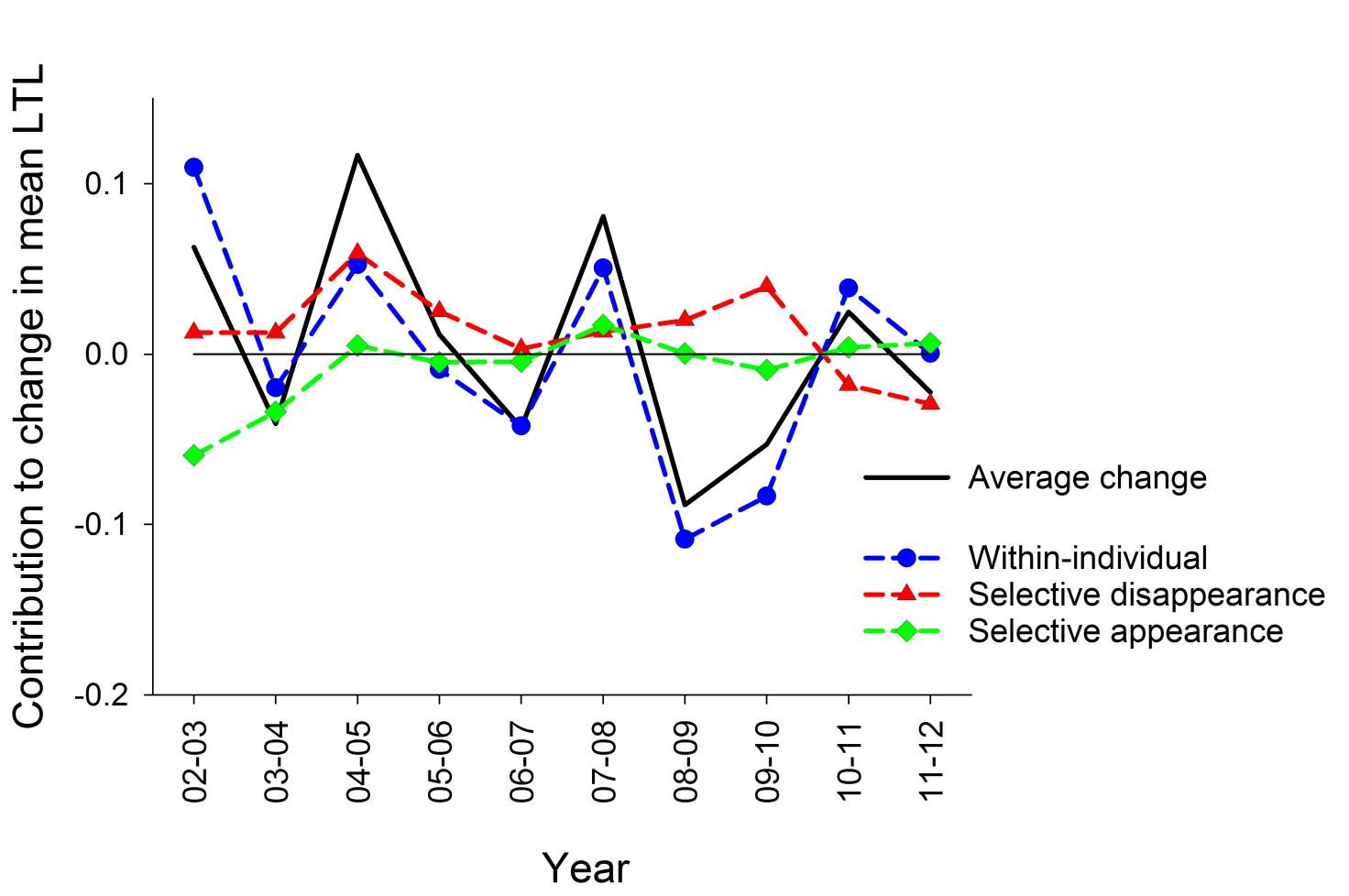


Figure S4. RLTL sample size by cohort and survival for (A) lambs measured in first August, (B) sheep sampled in August prior to 2004/2005 winter.


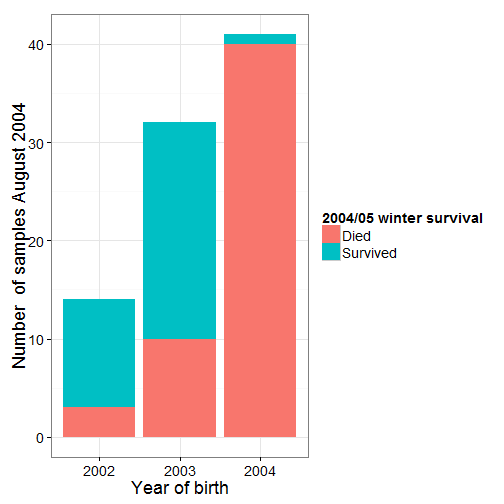

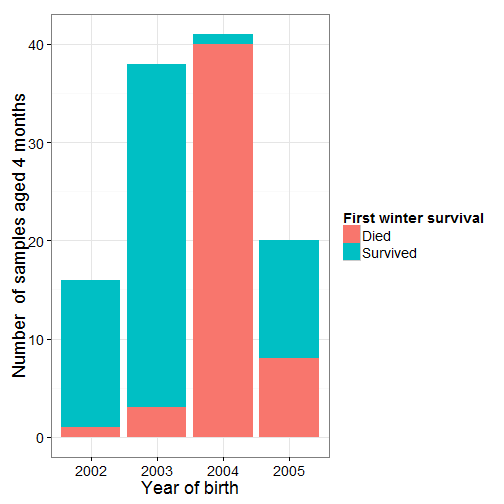


(B)

(A)

Figure S5. Correlation among telomere length measurements from 26 Soay sheep samples measured by both TRF and QPCR methods.

Table S1. Comparison of models of RLTL using AIC including different age functions (0 = no age function fitted, 1 = linear function, 2 = quadratic, 3 = cubic, F = factorial, T1 = single threshold, T2 = double threshold; age thresholds specified for threshold models) and birth cohort (four level factor) and its interaction with age. “K” denotes the number of terms in the model with models ordered with best fitting (lowest AIC) first and “Delta AIC” denoting difference between present and best fitting model.

| Age | | | Cohort | Cohort x age | K | AIC | Delta AIC |
| --- | --- | --- | --- | --- | --- | --- | --- |
| Function | Threshold 1 | Threshold 2 |  |  |  |  |  |
| F |  |  | Yes | Yes | 43 | -861.83 | 0 |
| T2 | 4 | 52 | Yes | Yes | 19 | -850.62 | 11.22 |
| T2 | 4 |  | Yes | Yes | 19 | -848.02 | 13.81 |
| T2 | 4 |  | Yes | Yes | 19 | -838.28 | 23.55 |
| T1 | 4 |  | Yes | Yes | 15 | -831.26 | 30.57 |
| T2 | 4 | 88 | Yes | Yes | 18 | -826.24 | 35.6 |
| T2 | 4 | 76 | Yes | Yes | 19 | -825.31 | 36.52 |
| F |  |  | No | No | 14 | -824.22 | 37.61 |
| F |  |  | Yes | No | 17 | -822.39 | 39.44 |
| T2 | 4 | 52 | No | No | 7 | -813.07 | 48.76 |
| T2 | 4 | 52 | Yes | No | 10 | -812.32 | 49.52 |
| T2 | 4 | 64 | No | No | 7 | -811.1 | 50.73 |
| T2 | 4 | 64 | Yes | No | 10 | -809.97 | 51.86 |
| T2 | 4 | 40 | No | No | 7 | -803.92 | 57.91 |
| T2 | 4 | 40 | Yes | No | 10 | -802.52 | 59.31 |
| T1 | 4 |  | No | No | 6 | -799.91 | 61.92 |
| T2 | 4 | 76 | No | No | 7 | -799.15 | 62.69 |
|  |  |  | Yes | No | 9 | -798.71 | 63.12 |
| T2 | 4 | 76 | Yes | No | 10 | -797.94 | 63.89 |
| T2 | 4 | 88 | No | No | 7 | -797.91 | 63.92 |
| T2 | 4 | 88 | Yes | No | 10 | -796.71 | 65.12 |
| T2 | 16 | 52 | Yes | Yes | 19 | -794.46 | 67.37 |
| T2 | 16 | 64 | Yes | Yes | 19 | -781.99 | 79.84 |
| T2 | 16 | 40 | Yes | Yes | 19 | -780.07 | 81.76 |
| 3 |  |  | Yes | Yes | 19 | -771.32 | 90.51 |
| T2 | 16 | 76 | Yes | Yes | 19 | -764.73 | 97.1 |
| T2 | 16 | 88 | Yes | Yes | 18 | -763.45 | 98.38 |
| T1 | 16 |  | Yes | Yes | 15 | -759.03 | 102.8 |
| T2 | 28 | 76 | Yes | Yes | 19 | -747.33 | 114.51 |
| T2 | 28 | 88 | Yes | Yes | 18 | -742.04 | 119.79 |
| T2 | 28 | 52 | Yes | Yes | 19 | -741.85 | 119.98 |
| T2 | 28 | 64 | Yes | Yes | 19 | -734.2 | 127.63 |
| 2 |  |  | Yes | Yes | 15 | -730.96 | 130.88 |
| T2 | 16 | 52 | Yes | No | 10 | -729.86 | 131.97 |
| Age | | | Cohort | Cohort x age | K | AIC | Delta AIC |
| Function | Threshold 1 | Threshold 2 |  |  |  |  |  |
| T2 | 28 | 40 | Yes | Yes | 19 | -728.43 | 133.4 |
| T2 | 16 | 52 | No | No | 7 | -727.25 | 134.58 |
| T2 | 16 | 64 | Yes | No | 10 | -726.78 | 135.05 |
| T2 | 16 | 64 | No | No | 7 | -725.46 | 136.37 |
| T2 | 28 | 52 | No | No | 7 | -723.96 | 137.87 |
| T2 | 28 |  | Yes | Yes | 15 | -721.64 | 140.2 |
| T2 | 16 | 40 | Yes | No | 10 | -720.87 | 140.96 |
| T2 | 28 | 64 | No | No | 7 | -719.39 | 142.45 |
| T2 | 16 | 40 | No | No | 7 | -719.02 | 142.81 |
| T1 | 16 |  | Yes | No | 9 | -717.91 | 143.92 |
| T1 | 16 |  | No | No | 6 | -717.87 | 143.96 |
| T2 | 28 | 64 | Yes | No | 10 | -716.84 | 144.99 |
| T2 | 16 | 76 | Yes | No | 10 | -716.59 | 145.24 |
| T2 | 16 | 76 | No | No | 7 | -716.35 | 145.48 |
| T2 | 16 | 88 | Yes | No | 10 | -715.93 | 145.9 |
| T2 | 16 | 88 | No | No | 7 | -715.91 | 145.92 |
| 3 |  |  | No | No | 7 | -714.94 | 146.89 |
| T2 | 28 | 40 | No | No | 7 | -714.61 | 147.23 |
| T2 | 28 | 40 | Yes | No | 10 | -713.22 | 148.61 |
| 3 |  |  | Yes | No | 10 | -712.6 | 149.23 |
| T2 | 28 | 76 | No | No | 7 | -708.91 | 152.92 |
| T2 | 28 | 88 | No | No | 7 | -708.43 | 153.4 |
| T1 | 28 |  | Yes | No | 9 | -707.04 | 154.79 |
| T2 | 28 | 76 | Yes | No | 10 | -705.67 | 156.17 |
| T2 | 28 | 88 | Yes | No | 10 | -705.07 | 156.76 |
| 2 |  |  | No | No | 6 | -702.33 | 159.51 |
| 1 |  |  | No | No | 5 | -698.7 | 163.13 |
| 2 |  |  | Yes | No | 9 | -697.79 | 164.04 |
| 1 |  |  | Yes | No | 8 | -694.72 | 167.11 |
| 1 |  |  | Yes | Yes | 11 | -694.11 | 167.72 |
| 0 |  |  | Yes | No | 7 | -693.28 | 168.55 |
| 0 |  |  | No | No | 4 | -689.41 | 172.42 |
| T1 | 28 |  | No | No | 6 | -551.94 | 309.9 |

Table S2. Comparison of models of RLTL excluding females with unknown longevity, using AIC including different age functions (0 = no age function fitted, 1 = linear function, 2 = quadratic, 3 = cubic, F = factorial, T1 = single threshold, T2 = double threshold; age thresholds specified for threshold models) and birth cohort (four level factor) and its interaction with age. “K” denotes the number of terms in the model with models ordered with best fitting (lowest AIC) first and “Delta AIC” denoting difference between present and best fitting model.

| Function | Threshold 1 | Threshold 2 | Cohort | Cohort x age | K | AIC | Delta_AIC |
| --- | --- | --- | --- | --- | --- | --- | --- |
| F |  |  | Yes | Yes | 43 | -805.41 | 0 |
| T2 | 4 | 52 | Yes | Yes | 19 | -794.49 | 10.91 |
| T2 | 4 | 64 | Yes | Yes | 19 | -792.3 | 13.11 |
| T2 | 4 | 40 | Yes | Yes | 19 | -782.8 | 22.61 |
| T1 | 4 |  | Yes | Yes | 15 | -776.31 | 29.09 |
| F |  |  | No | No | 14 | -776.28 | 29.13 |
| F |  |  | Yes | No | 17 | -773.83 | 31.57 |
| T2 | 4 | 88 | Yes | Yes | 18 | -771.13 | 34.28 |
| T2 | 4 | 76 | Yes | Yes | 19 | -770.36 | 35.05 |
| T2 | 4 | 52 | No | No | 7 | -766.36 | 39.04 |
| T2 | 4 | 52 | Yes | No | 10 | -764.38 | 41.03 |
| T2 | 4 | 64 | No | No | 7 | -764.26 | 41.15 |
| T2 | 4 | 64 | Yes | No | 10 | -761.98 | 43.43 |
| T2 | 4 | 40 | No | No | 7 | -757.5 | 47.9 |
| T2 | 4 | 40 | Yes | No | 10 | -754.99 | 50.42 |
| T1 | 4 |  | No | No | 6 | -753.33 | 52.07 |
| T2 | 4 | 76 | No | No | 7 | -752.57 | 52.84 |
| T2 | 4 | 88 | No | No | 7 | -751.33 | 54.07 |
| T1 | 4 |  | Yes | No | 9 | -750.94 | 54.47 |
| T2 | 4 | 76 | Yes | No | 10 | -750.2 | 55.21 |
| T2 | 4 | 88 | Yes | No | 10 | -748.94 | 56.47 |
| T2 | 16 | 52 | Yes | Yes | 19 | -742.95 | 62.46 |
| T2 | 16 | 40 | Yes | Yes | 19 | -730.86 | 74.54 |
| T2 | 16 | 64 | Yes | Yes | 19 | -730.55 | 74.86 |
| 3 |  |  | Yes | No | 19 | -719.03 | 86.38 |
| T2 | 16 | 76 | Yes | Yes | 19 | -712.03 | 93.38 |
| T2 | 16 | 88 | Yes | Yes | 18 | -711.46 | 93.95 |
| T1 | 16 |  | Yes | Yes | 15 | -708.38 | 97.02 |
| T2 | 28 | 76 | Yes | Yes | 19 | -695.58 | 109.83 |
| T2 | 28 | 52 | Yes | Yes | 19 | -693.18 | 112.23 |
| T2 | 28 | 88 | Yes | Yes | 18 | -690.49 | 114.92 |
| T2 | 28 | 64 | Yes | Yes | 19 | -685.9 | 119.51 |
| 2 |  |  | Yes | Yes | 15 | -684.1 | 121.31 |
| T2 | 16 | 52 | Yes | No | 10 | -683.72 | 121.69 |
| T2 | 16 | 52 | No | No | 7 | -683.18 | 122.23 |
| T2 | 16 | 64 | No | No | 7 | -681.32 | 124.09 |
| T2 | 16 | 64 | Yes | No | 10 | -680.83 | 124.58 |
| T2 | 28 | 52 | No | No | 7 | -680.2 | 125.21 |
| T2 | 28 | 40 | Yes | Yes | 19 | -679.76 | 125.65 |
| T2 | 28 | 52 | Yes | No | 10 | -677.49 | 127.92 |
| T2 | 28 | 64 | No | No | 7 | -675.73 | 129.68 |
| T2 | 16 | 40 | No | No | 7 | -675.25 | 130.15 |
| T2 | 16 | 40 | Yes | No | 10 | -675.16 | 130.25 |
| T1 | 16 |  | No | No | 6 | -674.09 | 131.31 |
| T1 | 28 |  | Yes | Yes | 15 | -674.05 | 131.36 |
| T2 | 16 | 76 | No | No | 7 | -672.55 | 132.86 |
| T1 | 16 |  | Yes | No | 9 | -672.37 | 133.04 |
| T2 | 16 | 88 | No | No | 7 | -672.14 | 133.26 |
| T2 | 28 | 64 | Yes | No | 10 | -672.06 | 133.35 |
| T2 | 28 | 40 | No | No | 7 | -671.5 | 133.91 |
| T2 | 16 | 76 | Yes | No | 10 | -671.05 | 134.36 |
| 3 |  |  | No | No | 7 | -670.87 | 134.53 |
| T2 | 16 | 88 | Yes | No | 10 | -670.39 | 135.02 |
| T2 | 28 | 40 | Yes | No | 10 | -668.67 | 136.74 |
| 3 |  |  | Yes | No | 10 | -667.3 | 138.11 |
| T1 | 28 |  | No | No | 6 | -667.15 | 138.26 |
| T2 | 28 | 76 | No | No | 7 | -665.64 | 139.77 |
| T2 | 28 | 88 | No | No | 7 | -665.2 | 140.21 |
| T1 | 28 |  | Yes | No | 9 | -662.7 | 142.7 |
| T2 | 28 | 76 | Yes | No | 10 | -661.32 | 144.09 |
| T2 | 28 | 88 | Yes | No | 10 | -660.73 | 144.67 |
| 2 |  |  | No | No | 6 | -658.99 | 146.42 |
| 1 |  |  | No | No | 5 | -655.42 | 149.99 |
| 2 |  |  | Yes | No | 9 | -653.81 | 151.6 |
| 1 |  |  | Yes | No | 8 | -651.24 | 154.16 |
| 1 |  |  | Yes | Yes | 11 | -650.48 | 154.92 |
| 0 |  |  | Yes | No | 7 | -650.08 | 155.32 |
| 0 |  |  | No | No | 4 | -646.46 | 158.95 |
